# MiR-10a as a Potential Biomarker and Therapeutic Target in Localized and Metastatic Prostate Cancer

**DOI:** 10.3390/cimb47110913

**Published:** 2025-11-03

**Authors:** Tiago José Borelli Bovo, Juliana Alves de Camargo, Ruan Pimenta, Vanessa Ribeiro Guimarães, Patrícia Candido, Katia Ramos Moreira Leite, Carlo Camargo Passerotti, William Carlos Nahas, Sabrina T. Reis

**Affiliations:** 1 Laboratório de Investigação Médica 55 (LIM55), Hospital das Clínicas HCFMUSP, Faculdade de Medicina, Universidade de São Paulo, São Paulo 01246903, SP, Brazilju.alvcam@gmail.com (J.A.d.C.);; 2 Department of Immunology and Immunotherapy, and Tisch Cancer Institute, Precision Immunology Institute, Icahn School of Medicine at Mount Sinai, New York, NY 10029, USA; 3 Uro-Oncology Group, Urology Department, Institute of Cancer State of São Paulo (ICESP), São Paulo 01246000, SP, Brazil

**Keywords:** prostate cancer, microRNAs, metastasis, invasion, proliferation, migrations, gene expression

## Abstract

**Introduction:** Prostate cancer (PC) accounts for around 10% of all cancers worldwide and is the fourth most common neoplasm. Localized PC has high cure rates when diagnosed early, but 35% of patients progress to the metastatic form. The search for new molecular markers, such as microRNAs, is fundamental to improving diagnosis and treatment. The role of miR-10a is controversial between tumor tissues, opening a niche for studies on their role in PC. **Objectives:** To evaluate the role of miR-10a in metastatic PC cell lines, focusing on the mechanisms of proliferation, migration, and invasion, and to analyze the expression in surgical specimens of localized PC. **Methods:** Three commercial metastatic PC cell lines were used: LNCaP, DU145 and PC-3. Expression of mimic miR-10a was induced by cell transfection, followed by extraction of miRNA and total RNA. The synthesis of complementary DNA (cDNA) and analysis by real-time PCR enabled the expression of miR-10a and the VEGF, MYC, and HAS3 genes to be assessed. Matrigel, colony formation, invasion, and migration assays were evaluated for the transfected cells. The surgical specimens were used to evaluate the miR-10a expression. **Results:** Transfected cells with mimic significantly increased the expression of miR-10a in the LNCaP (*p* = 0.0179), PC-3 (*p* ≤ 0.001), and DU145 (*p* ≤ 0.001) cell lines. Transfected cells reduced cell invasion in the PC-3 (*p* = 0.001) and DU-145 (*p* = 0.0004) cell lines and decreased cell migration and proliferation. In surgical specimens, miR-10a expression was higher in PC compared to Benign Prostatic Hyperplasia (*p* = 0.0010). **Conclusions:** Increased expression of miR-10a affects cell migration, invasion, and proliferation, showing potential as a therapeutic target in treating PC.

## 1. Introduction

Prostate Cancer (PC) represents around 7.3% of all cancer cases and is the fourth most common neoplasm in the world. In 2020, around 1.4 million new cases were diagnosed, corresponding to 15.2% of all types of cancer among men and an approximate risk of 31.50 cases per 100,000 men [1,2]. In Brazil, excluding non-melanoma skin tumors, it is the most incident malignant neoplasm in men, with an estimated 71,730 new cases between 2023–2025 and a risk of 67.86 cases per 100,000 men [3]. In 2022, 16,429 deaths were attributed to PC in Brazil [4,5]. Due to its high prevalence, PC represents a public health challenge, with factors such as screening, diagnosis, treatment, and mortality.

Malignant transformation of the prostate follows a multi-stage process, from prostatic intraepithelial neoplasia (PIN) to metastatic disease. PIN involves abnormal proliferation of cells in the prostatic ducts, which can develop into invasive cancer and subsequently spread to lymph nodes and distant organs, mainly bones, liver, and lungs [6]. In bone metastases, interaction with osteoblasts and osteoclasts favors tumor growth [7].

Although prostate-specific antigen (PSA) is widely used, the gold standard method for definitive diagnosis is transrectal ultrasound-guided prostate biopsy (BTRUS) [8]. Initially proposed by Donald Gleason [9], the Gleason system is widely used to define tumor aggressiveness [10]. When diagnosed early, PC has a better prognosis and is treated by radical prostatectomy or radiotherapy. In advanced cases, treatment is based on androgen suppression, but tumor heterogeneity makes lasting responses difficult [11,12].

MicroRNAs (miRNAs) are small non-coding RNAs that play a crucial role in regulating gene expression, influencing processes such as proliferation and apoptosis. miR-10a suppresses glioma and B-cell lymphoma [13,14], but its role in PC, especially metastatic PC, is controversial. Xiaoli et al. (2015) identified that miR-10a can influence disease progression, suggesting its potential as a prognostic biomarker [15]. Understanding its role in PC could open the possibility of new therapeutic approaches.

The search for new criteria or markers to improve patient risk classification and estimate the need for biopsy is ongoing, and molecular biology is one of the primary resources in this research. Therefore, this study aims to evaluate the effect of miR-10a stimulation on metastatic PC cell lines by analyzing its expression and that of its target genes in surgical specimens of PC. The selection of *VEGF*, *HAS3*, and *MYC* as target genes for analysis was based on prior evidence indicating their predicted binding sites for miR-10a and their well-established roles in pathways relevant to prostate cancer biology. *VEGF* is a critical mediator of angiogenesis, *HAS3* participates in hyaluronic acid synthesis and extracellular matrix remodeling, and *MYC* is a key oncogenic transcription factor involved in cell growth and proliferation. Together, these genes represent central nodes in tumor progression, making them biologically relevant candidates for assessing miR-10a regulatory effects in prostate cancer.

## 2. Materials and Methods

### 2.1. TCGA Cohort

The Cancer Genome Atlas (TCGA) is a publicly funded project that aims to catalog and discover the main genomic alterations that can cause cancer [16]. Using bioinformatics tools, we collected genomic data relating to prostate adenocarcinoma and adjacent normal prostate tissue, totaling 492 samples from PC and 52 paired normal tissue samples.

### 2.2. Prostate Cancer Cell Lines

Three commercial metastatic PC cell lines were used: LNCaP (lymph node metastasis), DU 145 (brain metastasis), and PC-3 (bone metastasis). These strains were obtained from the American Type Culture Collection (ATCC) and authenticated by the cell authentication service of the University of São Paulo (USP) Medical School. Depending on the cell line, the cells were grown in culture plates with MEM or RPMI media, supplemented with 10% fetal bovine serum and antibiotics. The culture conditions were adjusted to an atmosphere of 95% air and 5% CO2 at 37 °C.

### 2.3. Cell Transfection with miR-10a

To stimulate miR-10a expression, we used the miR-10a-5p mimic (synthetic microRNA) transfection technique. The cell transfection experiments were carried out in triplicate, with the miR-10a-5p mimic (MH10787) and the negative control mRNA precursor (Ambion, Austin, TX, USA). The cells were plated in 24-well plates and incubated with the transfection complex containing the RNAiMax agent Lipofectamine (Thermo Fisher Scientific, Waltham, MA, USA). After 48 h, the cells were collected for subsequent RNA extraction and functional analysis.

### 2.4. Extraction of Total RNA and miRNA

Following the manufacturer’s recommendations, total RNA and microRNAs were extracted using the miRVana^®^ microRNA Isolation Kit (Ambion, Austin, TX, USA). The purity and concentration of microRNAs and RNA were measured using a Nanodrop spectrophotometer^®^ (ND-1000, Wilmington, NC, USA) (260/280 nM).

### 2.5. Reverse Transcription (RT-qPCR)

The cDNA for microRNA analysis was synthesized using the TaqMan^®^ MicroRNA Reverse Transcription Kit (Applied Biosystems, Foster City, CA, USA), while the cDNA for gene analysis was synthesized using the High-Capacity cDNA Reverse Transcription^®^ kit. The qPCR was carried out on the ABI 7500 Fast RT-PCR system using TaqMan^®^ probes. The expression of the target genes (VEGF, MYC, HAS3) was normalized by the reference gene B2M, and RNU48 normalized the expression of miR-10a. The data was analyzed using the 2-^ΔΔCt^ method.

### 2.6. Matrigel Invasion Assay

The invasion assay was carried out using bio Coat Matrigel Invasion Chambers (Becton Dickinson, Bedford, MA, USA) containing 8 μm membranes coated with Matrigel. Cells transfected with miR-10a were plated on the inserts and incubated for 48 h. After incubation, the non-invasive cells were removed, while the migrated cells were fixed, stained with crystal violet, and counted in nine microscopic fields (200×).

### 2.7. Migration Assay

The wound healing assay was carried out to assess cell migration [14]. The cells were plated in 24-well plates, and after reaching confluence, a 200 µL tip was used to streak the cells. The cells were then transfected with miR-10a and photographed at 0, 24, and 48 h. Migration was analyzed using ImageJ software (version v.1.53k). U. S. National Institutes of Health, Bethesda, MD, USA.

### 2.8. Proliferation Test

The cells were plated at low density (5 × 10^2^ cells/well) in 12-well plates and transfected with miR-10a. After the incubation period, the cells were quantified to analyze the effect of miR-10a on cell proliferation. Colonies smaller than 1 mm were not counted. The plates were photographed and the images were analyzed using the ImageJ software (version v.1.53k) W.S., ImageJ, U. S. National Institutes of Health, Bethesda, MD, USA.

### 2.9. Collection and Processing of Samples

Ten surgical specimens from patients with localized PC treated at the Sírio-Libanês Hospital were selected to analyze the expression profile of miR-10a and the target genes. As a control, five benign prostate tissue samples of hyperplastic glands from patients also operated on at the same institution were used. The samples were stored in paraffin blocks in the Medical Research Laboratory 55 (LIM 55). All of the patients signed an informed consent form, and the study was approved by the Ethics Committee of the USP School of Medicine (opinion no. 4.294.500). MicroRNA and total RNA were extracted from the tissues using the miRVana^®^ microRNA kit (Ambion, Austin, TX, USA), following the manufacturer’s guidelines. The process involved addition of a lysis buffer, maceration with Tissue Lyser LT, addition of a homogenization additive, and incubation of the samples on ice.

These steps were followed by extraction, reverse transcription and real-time PCR, as per the established protocol.

### 2.10. Statistical Analysis

The data was presented as mean and standard deviation for the quantitative variables. The groups were compared using Student’s *t*-test and ANOVA (homogeneous variables), and Mann–Whitney (non-homogeneous variables). SPSS19.0 software was used for statistical analysis. A significance level of 5% was adopted for all analyses, i.e., results with a *p*-value of less than 5% (*p* < 0.05) were considered statistically significant.

## 3. Results

### 3.1. Bioinformatics Analysis

**Figure 1:**
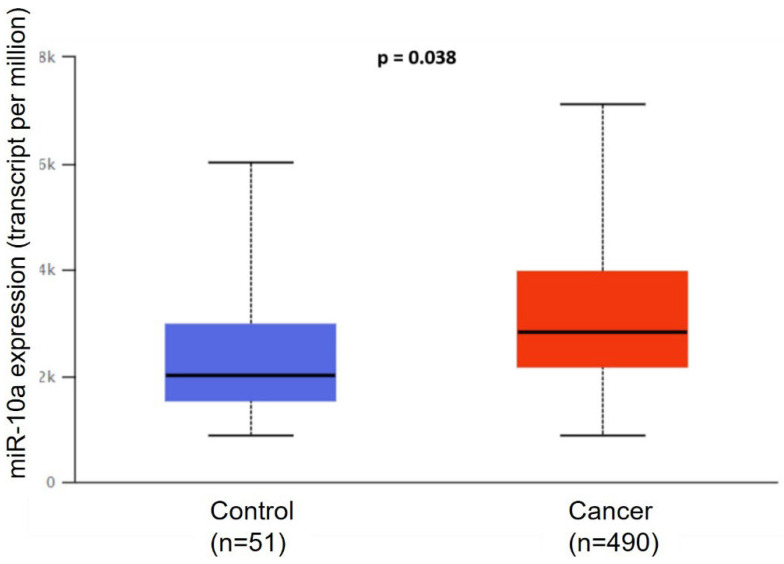
Analysis of miR-10a expression in prostate cancer. Expression levels of miR-10a in PC and normal prostate tissues were analyzed using TCGA (The Cancer Genome Atlas) database. Data are presented as mean ± SD.

Bioinformatics analysis in the TCGA program showed that miR-10a is overexpressed in the cancer group (*n*= 490) compared to the control (*n* = 51) (*p* = 0.038) (Figure 1).

### 3.2. Cell Transfection in Prostate Cancer Lines

**Figure 2:**
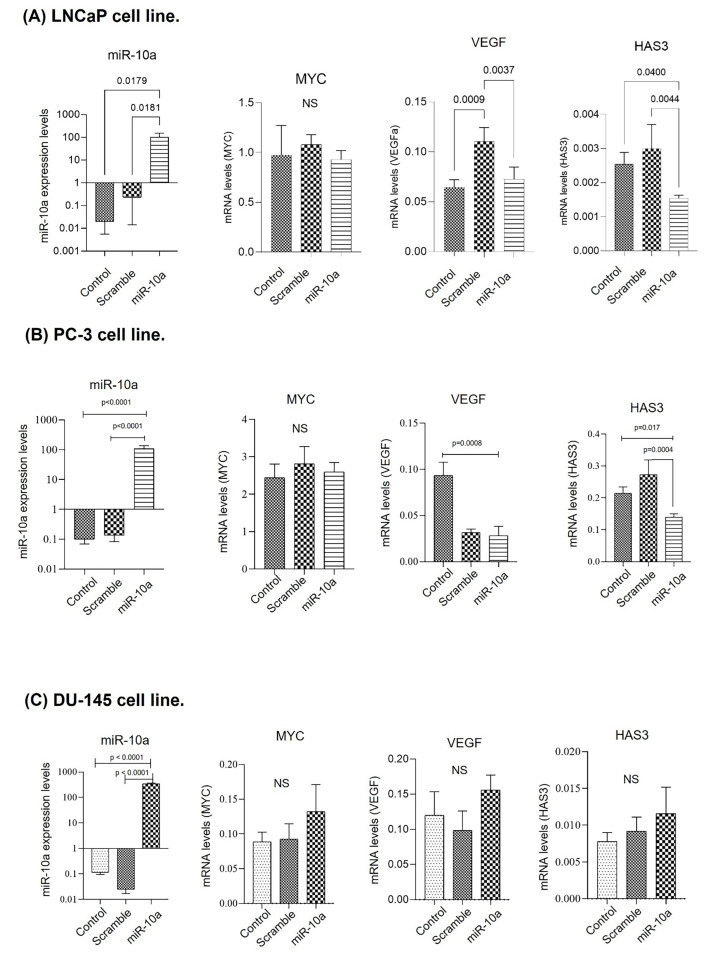
Expression of miR-10a and predicted target genes in prostate cancer cell lines. Relative expression of miR-10a and its target genes was evaluated in (**A**) LNCaP, (**B**) PC3, and (**C**) DU-145 cells compared to non-transfected controls. Gene expression was quantified by RT-qPCR and normalized to endogenous controls (NS = not significant).

Cell transfection was performed on the LNCaP, PC-3, and DU-145 cell lines. In the LNCaP cell line, the expression of miR-10a was increased when compared to the control (*p* = 0.01) after transfection, demonstrating the technique’s success. VEGF and HAS3 showed reduced expression (*p* = 0.003; *p* = 0.004 respectively), suggesting that they are target genes of miR-10a in this cell line. The MYC gene showed no significant change (Figure 2A). The results of the PC-3 cell line were similar to those of the LNCaP cells, with increased expression of miR-10a when compared to the control group (*p* < 0.0001), along with reduced expression of VEGF and HAS3 (*p* = 0.0008; *p* = 0.017 respectively) but not MYC (Figure 2B). With the DU-145 cells, despite the success of the transfection, there was no significant reduction in the expression of the target genes (Figure 2C).

### 3.3. Cell Invasion Experiment (Matrigel)

**Figure 3:**
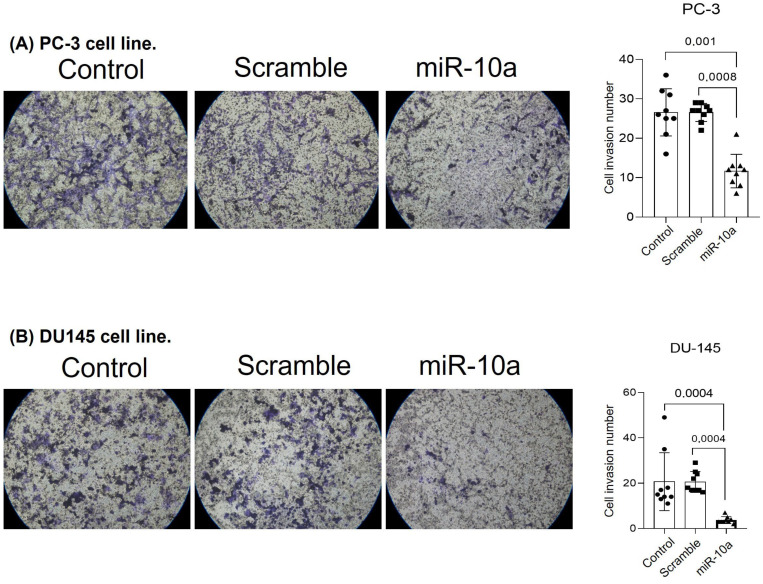
Invasion assay following miR-10a transfection. Representative images and quantification of Matrigel invasion assays in (**A**) PC3 and (**B**) DU-145 cells after transfection with miR-10a mimics or negative control. (Samples: circles: Control; squares: Scramble; triangles: miR-10a).

We evaluated if transfection with miR-10a would affect the tumor invasion capacity of PC-3 and DU-145 cells. Cells transfected with miR-10a showed a reduced ability to invade the basal membrane simulated by Matrigel. This effect was observed in the PC-3 (*p* < 0.001) (Figure 3A) and DU-145 (*p* = 0.0004) (Figure 3B) cell lines.

### 3.4. Migration Experiment

**Figure 4:**
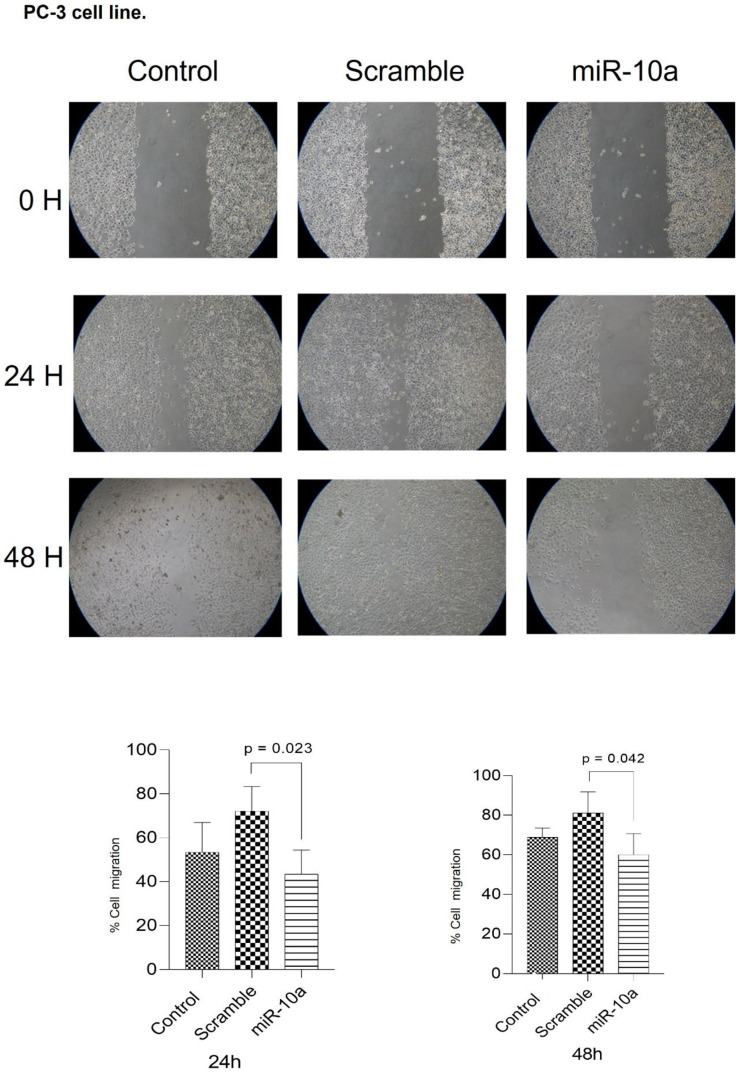
Cell migration assay in PC3 cells after miR-10a overexpression. Wound-healing assay showing the migratory capacity of PC3 cells transfected with miR-10a mimics compared to control groups at 24 and 48 h. The wound area was measured at each time point, and migration was quantified using ImageJ software (NIH, Bethesda, MD, USA).

**Figure 5:**
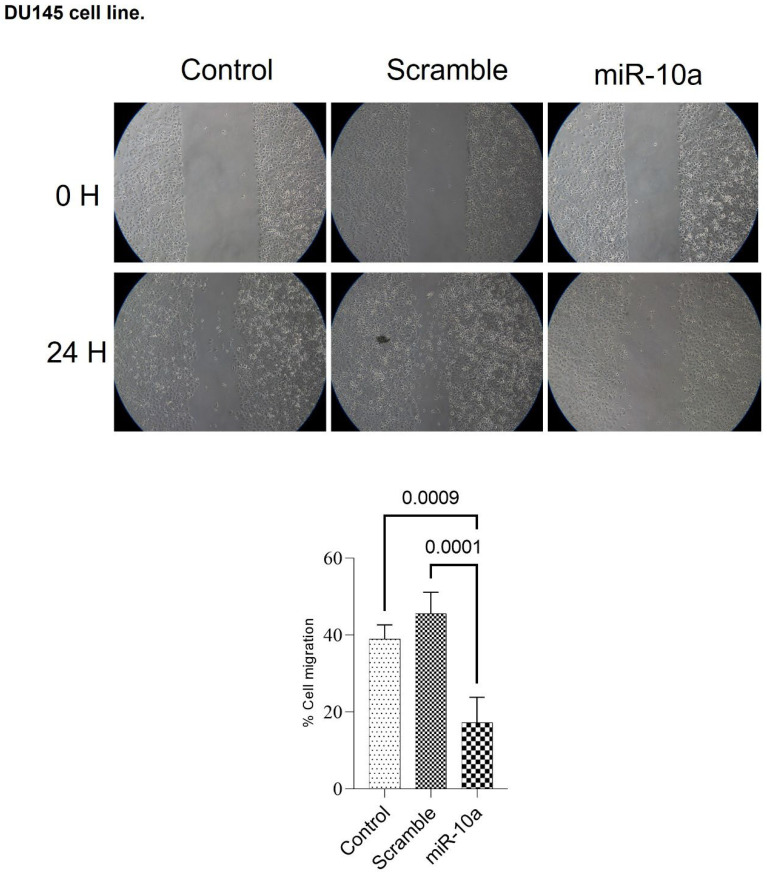
Cell migration assay in DU-145 cells after miR-10a transfection. Wound-healing assay demonstrating the effect of miR-10a overexpression on the migratory capacity of DU-145 cells at 24 h compared to controls. The wound closure area was quantified using ImageJ software (NIH, Bethesda, MD, USA).

We analyzed the impact of miR-10a transfection on cell migration, calculating the area without cells during the treatment in culture plates after 24 and 48 h. For the PC-3 cells, there was a reduction in the migration potential of cells transfected with miR-10a compared to controls, both at 24 (*p* = 0.023) and 48 (*p* = 0.042) hours (Figure 4). With the DU-145 cells, migration was reduced at 24 h (*p* = 0.0001), but could not be assessed at 48 h, as the cells in the control group had completely closed the area, preventing comparison (Figure 5).

### 3.5. Cell Proliferation Experiment (Colony Formation)

**Figure 6:**
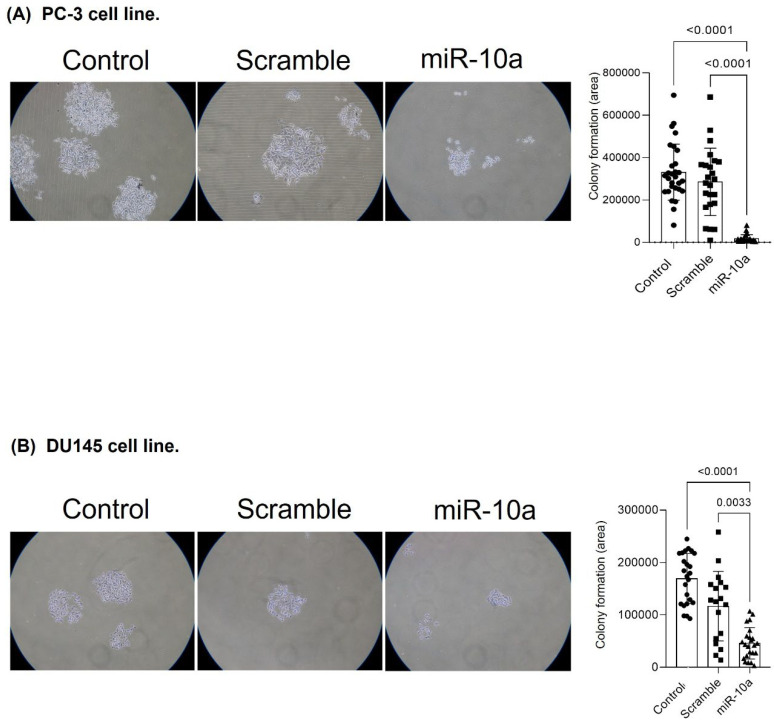
Effect of miR-10a on cell proliferation. Colony formation assay showing proliferation of (**A**) PC3 and (**B**) DU-145 cells after miR-10a transfection compared to control groups. Cells were plated at low density (5 × 10^2^ cells/well), and after incubation, colonies larger than 1 mm were fixed, stained with crystal violet, and counted using ImageJ software (Samples:. circles: Control; squares: Scramble; triangles: miR-10a).

We evaluated the influence of miR-10a on cell proliferation through colony formation. In the PC-3 cells, transfection with miR-10a reduced prolife ration potential, resulting in smaller colonies (*p* < 0.001) (Figure 6A). For the DU-145 cells, we observed similar results, with a reduction in the number and size of colonies in cells transfected with miR-10a (*p* < 0.001) (Figure 6B).

### 3.6. Expression of miR-10a and Target Genes in Surgical Specimens

**Figure 7:**
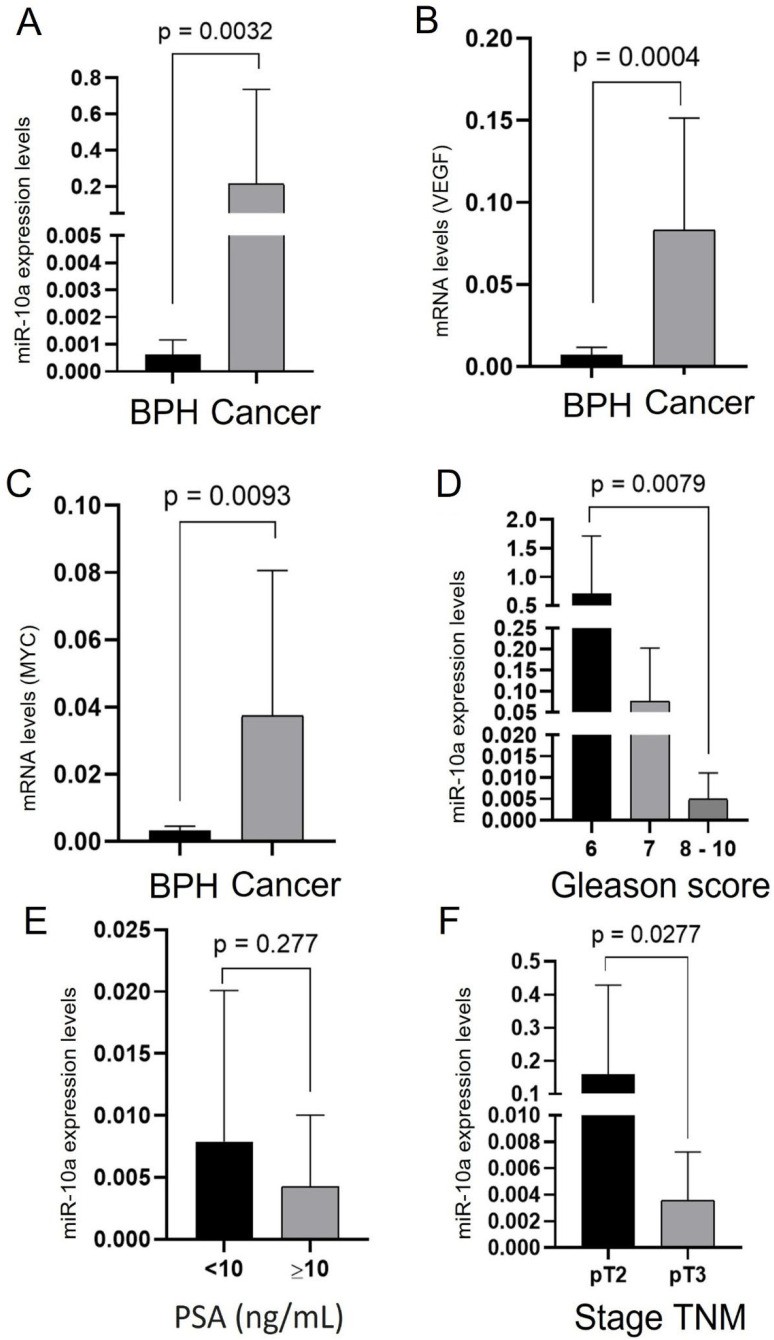
Expression analysis of miR-10a and target genes MYC and VEGF in patient samples. (**A**) miR-10a expression in BPH and primary PC tissues. (**B**) VEGF gene expression in BPH and PC samples. (**C**) MYC gene expression in BPH and PC samples. (**D**) Comparison of miR-10a expression among Gleason score groups (6–7, and 8–10). (**E**) miR-10a expression according to PSA levels (<10 vs. ≥10 ng/mL). (**F**) miR-10a expression between TNM stages pT2 and pT3.

In the second part of the study, we analyzed the expression of miR-10a, MYC, and VEGF genes in tissue samples from primary PC (n = 10). We used tissue samples from benign prostatic hyperplasia (BPH) (n = 5) as a control group. Real-time PCR analysis showed that although miR-10a expression was low in both groups, patients with PC showed higher microRNA expression (*p* = 0.0032). Concerning the genes evaluated, the VEGF and MYC genes were overexpressed in the PC samples compared to the control group (*p* = 0.0004 and *p* = 0.0093 respectively) (Figure 7A–C). HAS3 could not be analyzed due to the lack of amplification in the samples. We also assessed the relationship between miR-10a expression and the patients’ clinical and pathological data. We found a higher expression of miR-10a with more favorable characteristics, such as Gleason score 6 (*p* = 0.0079) and pathological staging pT2 (*p* = 0.0277). No differences in PSA analyses between groups (*p* = 0.277) (Figure 7D–F) were determined.

## 4. Discussion

In this study, we investigated the role of miR-10a in the development and tumorigenic processes in prostate cancer tissue. Analysis of TCGA data showed that miR-10a is overexpressed in PC samples compared to control samples, a finding confirmed by our surgical specimens. This result is important because the literature on the role of miR-10a in cancer progression is varied. In some cases, miR-10a acts as an oncomiR, while in others it acts as a tumor suppressor, depending on the type of cancer and the cellular context. In lung cancer, for example, it decreases the expression of the PTEN gene, promoting tumor progression [17]. The same mechanism was described in the study by Zeng et al. (2014) with cervical cancer [18]. However, miR-10a is described as a tumor suppressor in glioma, ovarian cancer, and diffuse large B-cell lymphoma [19,20,21]. Xiaoli Z. et al. (2015) found that miR-10a is underexpressed in PC tumor tissues, suggesting its potential as a prognostic and predictive marker [15].

Despite the apparent discrepancy between our in vitro and clinical findings, this dual behavior of miR-10a has also been reported in other malignancies, suggesting a context-dependent regulatory function. While in vitro overexpression of miR-10a reduced invasion, migration, and proliferation, indicating a tumor suppressive role, its upregulation in clinical prostate cancer tissues may reflect compensatory mechanisms within the tumor microenvironment. The tumor milieu, characterized by altered stromal signaling, inflammatory mediators, and epigenetic reprogramming, can modify miRNA activity and target interactions. Therefore, it is plausible that miR-10a exerts differential effects depending on the cellular and molecular context, acting as a tumor suppressor in controlled experimental conditions while being upregulated in response to oncogenic signaling in vivo. This hypothesis is consistent with the literature describing miR-10a as both an oncomiR and a tumor suppressor in a tissue-specific manner [17,18,19,20,21].

To further elucidate the biological mechanisms underlying these observations, we examined the potential downstream pathways associated with the identified target genes VEGF, HAS3, and MYC. VEGF and HAS3 are key regulators of angiogenesis and extracellular matrix organization, both crucial for PC progression. The observed downregulation of these genes after miR-10a overexpression suggests a possible inhibitory effect on angiogenic and migratory pathways. Although no significant modulation of MYC expression was detected in our in vitro assays, MYC overexpression in clinical samples supports its established oncogenic role in prostate cancer [22,23,24,25,26,27]. Together, these findings indicate that miR-10a may exert a multifaceted regulatory influence on PC biology, interacting with angiogenic and proliferative signaling cascades in a manner that depends on tumor stage, cell type, and microenvironmental cues.

We observed that overexpression of miR-10a reduced the expression of the VEGF and HAS3 genes in the LNCaP and PC-3 cell lines and decreased cell invasion and migration after 24 and 48 h. These data suggest that miR-10a acts as a tumor suppressor in PC. It may be involved in mechanisms such as the downregulation of KDM4A and cell growth suppressors, indicating its potential as a therapeutic target [28]. Our results elucidate the potential use of miR-10a as a prognostic marker in PC and a viable therapeutic target for future clinical interventions.

Transfection of miR-10a in the LNCaP, PC3 and DU145 cell lines significantly increased the expression of this miRNA, confirming the efficacy of transfection. However, we observed variations in the effects on the VEGF, HAS3, and MYC target genes. In the LNCaP and PC3 cell lines, overexpression of miR-10a resulted in lower expression of VEGF and HAS3, but there were no differences in MYC expression. A previous study showed that overexpression of miR-10a in MC3T3-E1 cells and MUVECs reduced VEGF and VE-cadherin levels, indicating its negative regulation of these angiogenic factors and, consequently, the suppression of new blood vessel formation [29]. In another study, HAS3 was identified as a direct target of miR-10a, confirming its negative regulation [30]. These results suggest that VEGF and HAS3 may be direct targets of miR-10a, in spite of there being no data in the literature for PC samples.

Although no significant reduction in MYC gene expression was observed after miR-10a transfection in the cell lines, this proto-oncogene is believed to be associated with the progression of PC [22], with its overexpression related to the development of the disease [23,24]. Analysis of the surgical specimens showed that the MYC gene is overexpressed in the PC samples, although at low levels compared to the control group, suggesting a compensatory or regulatory role in the tumor environment. Previous studies indicate that MYC amplification in PC occurs in 10–30% of localized prostate tumors and more than 50% of advanced tumors, associated with a worse prognosis [25,26]. Several studies in the literature analyze MYC expression in PC by immunohistochemistry, while this study analyzed miRNA gene expression. The overexpression of MYC in PC samples reinforces its role as an oncogene in the progression of PC, which is frequently overexpressed in most cancers, especially in somatic genetic alterations such as translocations and gene amplifications [22]. In addition, it regulates intrinsic cancer cell pathways, promoting growth and survival, such as proliferation, metabolism, invasion, autophagy and protein biosynthesis. Its activation also drives angiogenesis, contributing to tumor progression [27].

Our experiments with the PC-3 and DU-145 cell lines showed that after transfection with miR-10a, there was a significant reduction in the invasive capacity of these cells in the matrigel layer. This suggests that miR-10a may be necessary in limiting cell invasion and metastasis formation. Feiying Gao et al. (2024) showed that miR-10a inhibited migration and invasion in ovarian cancer cells by negatively regulating the transcription factor GATA6, an oncogenic protein [20]. This effect can be explained by the negative regulation of miR-10a in genes involved in extracellular matrix degradation and cell motility. Yankun Liu et al. (2017) also support this hypothesis, showing that miR-10a can inhibit colorectal cancer metastasis by regulating epithelial–mesenchymal transition, an apoptosis process induced by loss of cell adhesion. In addition, miR-10a can affect the expression of matrix metalloproteinases (MMPs), enzymes that degrade extracellular matrix components, facilitating tumor invasion [31]. In summary, our results suggest that miR-10a may suppress cell invasion in PC, limiting tumor cells’ aggressiveness and metastatic capacity.

We evaluated the effect of miR-10a overexpression on cell migration using the migration assay in PC-3 and DU-145 cell lines. Both cells transfected with miR-10a showed a decrease in migratory capacity compared to control cells, which were not treated with the miRNA. This reduction can be explained by the negative regulation of the HAS2 and HAS3 genes, which are responsible for the synthesis of hyaluronic acid (HA) [32]. When overexpressed, HA, the main component of the extracellular matrix, contributes to tumor neovascularization by attracting stromal cells such as fibroblasts, monocytes/macrophages, endothelial cells, stem cells, and mesenchymal cells, promoting tumor growth [33]. In PC, the accumulation of HA in the tumor microenvironment, mediated by the HAS2 gene, is associated with higher migration, tumor growth, angiogenesis, and a worse prognosis [33,34]. The study by Liu N. et al. (2001) associates the overexpression of HA, induced by the expression of HAS3, with the process of angiogenesis [35]. Furthermore, although the experimental limitation in the DU-145 cell line prevented the measurement of migration at 48 h due to the complete closure of the wound in the control group, we suggest that miR-10a has a consistent inhibitory effect on cell migration, reinforcing its potential as a tumor suppressor in PC.

Regarding cell proliferation, our colony formation assays showed that transfection of miR-10a into the PC-3 and DU-145 cell lines reduced the number and size of colonies formed. This finding is consistent with the study by Mu H. et al. (2019), who also observed limited colony formation after miR-10a transfection in the LNCaP and PC3 cell lines [28]. These results indicate that overexpression of miR-10a is associated with a lower potential for colony formation, suggesting that miR-10a may be a tumor suppressor in metastatic PC.

We also observed a relationship between miR-10a expression and favorable clinical characteristics in patients with PC. Higher miR-10a expression was associated with lower disease aggressiveness, such as Gleason score 6 and pathological stage pT2. This was identified by Mu H. et al. (2019), where lower miR-10a expression was associated with higher Gleason scores [28]. We found no data confirming the association between PSA and pathological stages. Although miR-10a displayed tumor-suppressive properties in our functional assays, its therapeutic potential should be interpreted within this biological context. Strategies aimed at restoring or mimicking miR-10a expression in tumors where it is downregulated could represent a promising therapeutic avenue. In this sense, miR-10a functions as a molecular candidate for replacement therapy rather than direct inhibition, similar to other tumor-suppressive microRNAs currently being explored in preclinical studies.

In addition, we explored the availability of miR-10a expression data in other repositories, such as cBioPortal and COSMIC, to complement our TCGA-based analysis. However, the limited availability and inconsistency of prostate cancer–specific miRNA datasets across these platforms restricted additional comparisons. For this reason, only TCGA data were used, ensuring methodological consistency and reliability. Considering the tissue samples used, one limitation of our study is the small number of patients sampled. Future studies should increase the sample size, use in vivo models, and explore different types of samples and experimental conditions.

## 5. Conclusions

Our study showed that the expression of miR-10a in PC is significantly higher than in BPH tissues, a finding also confirmed in bioinformatic analyses and surgical specimens. We believe that miR-10a is associated with tumor progression mechanisms such as migration, invasion, and cell proliferation, and may act as a tumor suppressor in PC. However, more research is needed to confirm these findings and explore the therapeutic and prognostic potential of miR-10a in PC.

## Data Availability

The original contributions presented in this study are included in the article. Further inquiries can be directed to the corresponding author.
